# Supplementary material for: The causal relationship between the human gut microbiota and pyogenic arthritis: a Mendelian randomization study
Source: Front Cell Infect Microbiol. 2024 Nov 26;14:1452480. doi: 10.3389/fcimb.2024.1452480 (PMC11629706; doi:10.3389/fcimb.2024.1452480)
Supplement: Supplementary file 1 [file DataSheet1.zip › mendelian test/ebi-a-GCST90027522.csv_forest.pdf]

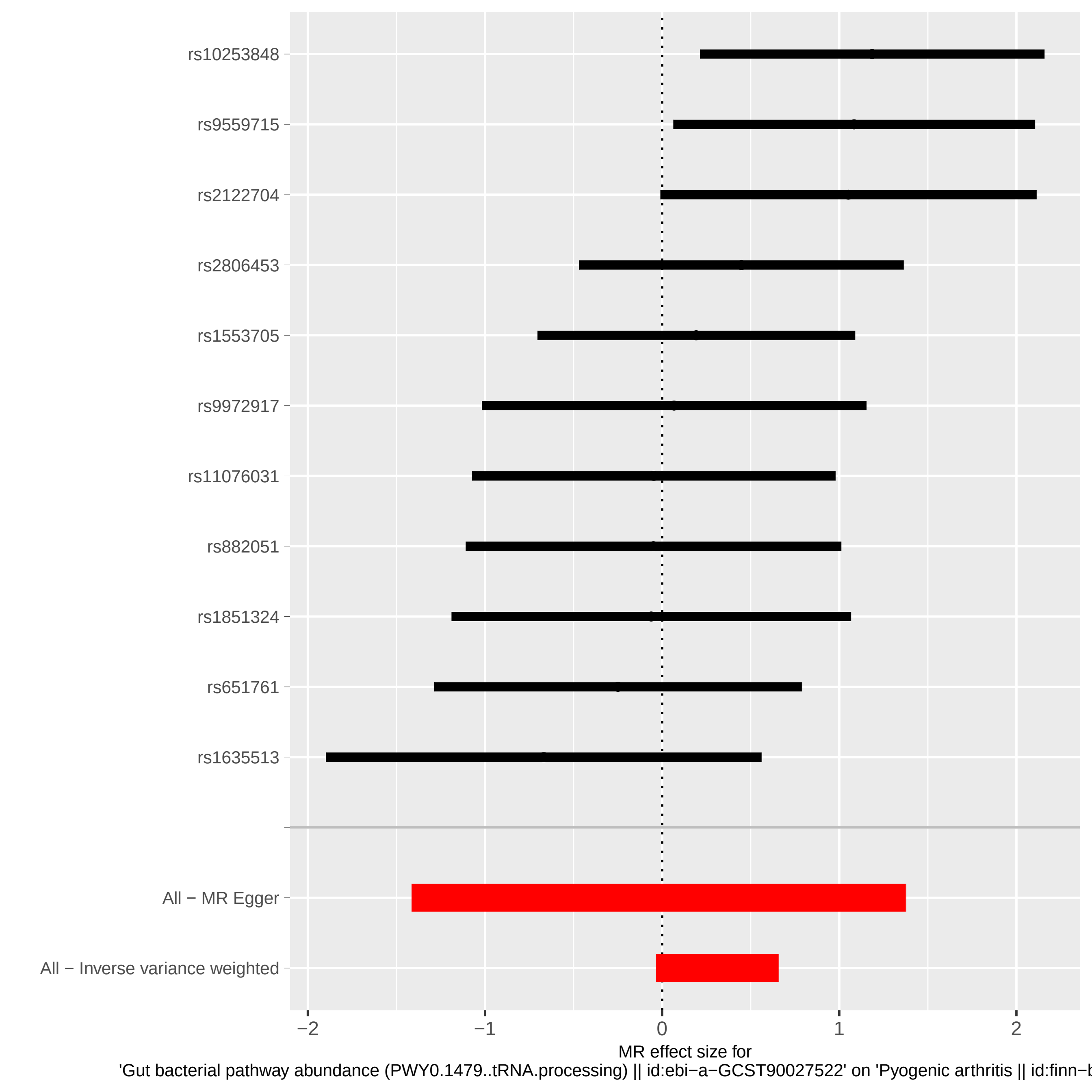
rs10253848
rs9559715
rs2122704
rs2806453
rs1553705
rs9972917
rs11076031
rs882051
rs1851324
rs651761
rs1635513
All − MR Egger
All − Inverse variance weighted
−2
−1
0
1
2
MR effect size for
'Gut bacterial pathway abundance (PWY0.1479..tRNA.processing) || id:ebi−a−GCST90027522' on 'Pyogenic arthritis || id:finn−b
